# Inhibition of Drosophila Wg Signaling Involves Competition between Mad and Armadillo/β-Catenin for dTcf Binding

**DOI:** 10.1371/journal.pone.0003893

**Published:** 2008-12-09

**Authors:** Yi Arial Zeng, Maryam Rahnama, Simon Wang, Wendy Lee, Esther M. Verheyen

**Affiliations:** Department of Molecular Biology and Biochemistry, Simon Fraser University, Burnaby, British Columbia, Canada; Katholieke Universiteit Leuven, Belgium

## Abstract

Precisely regulated signal transduction pathways are crucial for the regulation of developmental events and prevention of tumorigenesis. Both the Transforming Growth Factor β (TGFβ)/Bone morphogenetic protein (BMP) and Wnt/Wingless (Wg) pathways play essential roles in organismal patterning and growth, and their deregulation can lead to cancers. We describe a mechanism of interaction between Drosophila Wg and BMP signaling in which Wg target gene expression is antagonized by BMP signaling. *In vivo*, high levels of both an activated BMP receptor and the BMP effector Mad can inhibit the expression of Wg target genes. Conversely, loss of *mad* can induce Wg target gene expression. In addition, we find that ectopic expression in vivo of the Wg transcription factor dTcf is able to suppress the inhibitory effect caused by ectopic Mad. In vitro binding studies revealed competition for dTcf binding between Mad and the Wnt effector β-catenin/Armadillo (Arm). Our in vivo genetic analyses and target gene studies support a mechanism consistent with the in vitro binding and competition studies, namely that BMP pathway components can repress Wg target gene expression by influencing the binding of Arm and dTcf.

## Introduction

Wnt/Wingless (Wg) and TGFβ/BMP are two distinct families of secreted ligands that employ different signaling components to exert their biological effects. These pathways play essential roles in the growth and patterning of most tissues across species from worms to humans (reviewed in [1], [2]). Therefore, any insight that can be gained into their interactions may reveal important mechanisms of regulation that participate in fine-tuning signaling and achieving proper differentiation. In addition, mutations in components of these pathways can have dire consequences for organismal development and survival. Thus, identifying regulatory networks can provide insight into aberrant development and cancers that result when regulation is disrupted.

Wg signaling is mediated by the DNA-binding transcription factor, dTcf, and its co-activator β-catenin/Armadillo (Arm) [3]. In the absence of Wg signaling, cytoplasmic Arm levels are kept low through continuous proteasome-mediated degradation, which is controlled by a complex of Zw3, APC and Axin [4], [5], [6], [7]. Wg signaling results in down-regulation of Zw3 kinase activity, which allows Arm to escape degradation and accumulate in the cytoplasm. Subsequently, Arm proceeds into the nucleus where it forms a complex with dTcf, a member of the lymphoid enhancer factor 1 (Lef)/T-cell factor (Tcf) family of transcription factors. Arm and dTcf regulate the transcriptional activation of numerous target genes [3], [8], [9]. These genes, such as *achaete (ac)*, *senseless* (sens) and *distal-less* (*dll*) organize the D/V boundary of the wing and contribute to adult features such as the wing margin [10], [11].

The best-characterized TGFβ ligand in Drosophila is Decapentaplegic (Dpp), a member of the BMP family of ligands (reviewed in [12]). Upon Dpp ligand binding and receptor oligomerization, the type II receptor Punt activates the serine/threonine kinase activity of the type I receptor Thickveins (Tkv). Tkv then phosphorylates the receptor-regulated Smad, Mothers against Dpp (Mad), which then allows it to bind the co-Smad Medea (Med), translocate to the nucleus and bind DNA to activate gene expression.

The Drosophila wing has served as a very amenable tissue in which to dissect the roles of signaling molecules in development. Adult wing patterning is initiated in epithelial cells of the larval imaginal discs. In wing disc development, the Wingless (Wg) member of the Wnt family organizes the dorsal/ventral (D/V) axis, while BMPs are is required to pattern the anterior/posterior (A/P) axis (reviewed in [13]). Both pathways regulate the expression of target genes that influence wing shape, size and patterning. Consistent with these diverse roles in the wing, mutations in the two pathways result in distinct adult wing phenotypes. Reduced Wg signaling leads to loss of the entire wing blade, or loss of D/V wing margin tissue, resulting in variable notching [14]. Reduced BMP leads to the formation of wings that are typically larger than wild-type and have vein patterning defects (reviewed in [15]).

Crosstalk between Wnt/Wg and TGFβ/BMP signaling pathways has been extensively described in numerous systems. These interactions involve both cooperation and antagonism between these pathways to modulate gene expression. This cross regulation has generally focused on the role of both pathways in regulation of transcription. It has been previously shown that vertebrate Lef/Tcf proteins can associate with Smads and synergistically activate transcription of *Xtwn* [16], [17] and mouse *c-myc* [18]. In Drosophila, the expression of several genes is regulated coordinately by Dpp and Wg, including *Ubx* and *dpp* [19], [20], [21], [22], [23]. Furthermore, a mutual antagonism between Wg and Dpp pathways that results in transcriptional repression of the two ligands, *wg* and *dpp,* has been well described in the Drosophila leg imaginal disc. Ectopic Dpp signaling in leg discs leads to reduced *wg* ligand expression and phenotypes indicative of loss of Wg signaling. Conversely, ectopic Wg expression leads to a loss of Dpp ligand and phenotype associated with loss of Dpp signaling [24], [25], [26]. However, it has been noted in numerous studies that a different mechanism exists in the wing disc, as altering the individual pathways does not affect the ligand expression [24], [25]. Ectopic Dpp signaling in the wing pouch does not result in changes in *wg* expression, yet it can cause phenotypes indicative of loss of Wg signaling, such as wing notching [27]. Another example of their different roles in wing development compared to the leg is seen with the regulation of Dll. In the leg, *dll* expression requires input from both Wg and Dpp [28], [29], while in the wing the expression of *dll* depends only on input from the Wg pathway [11], [30].

We and others have noticed wing patterning phenotypes that occur upon modulation of Wg and Dpp pathways that suggest a level of cross-regulation and we sought to examine this more carefully. In this study, we describe a regulatory interaction in the *Drosophila* wing imaginal disc between Wg and BMP signaling. We show that in vivo BMP signaling can inhibit Wg pathway activity. Genetic interactions and phenotypic observations reveal elevated Wg signaling in the absence of BMP activity and conversely, reduced Wg signaling is found upon elevated BMP signaling. Clonal analyses in the larval wing disc reveal that expression of Wg target genes is repressed by ectopic BMP signaling. Conversely, loss of endogenous Mad leads to ectopic expression of the Wg target Dll. In cell culture experiments we find that Arm and Mad compete for association with dTcf. While ectopic expression of dTcf does not enhance the output of the Wg pathway [3], we found that it could suppress the effects of ectopic Mad. These data suggest that in vivo, excess dTcf can titrate the effects of ectopic Mad, supporting our competition model. Taken together, our results suggest a mechanism by which Mad represses Wg target genes by influencing the association of Arm and dTcf.

## Results

### Ectopic Dpp signaling can mimic Wg loss of function phenotypes

**Figure 1:**
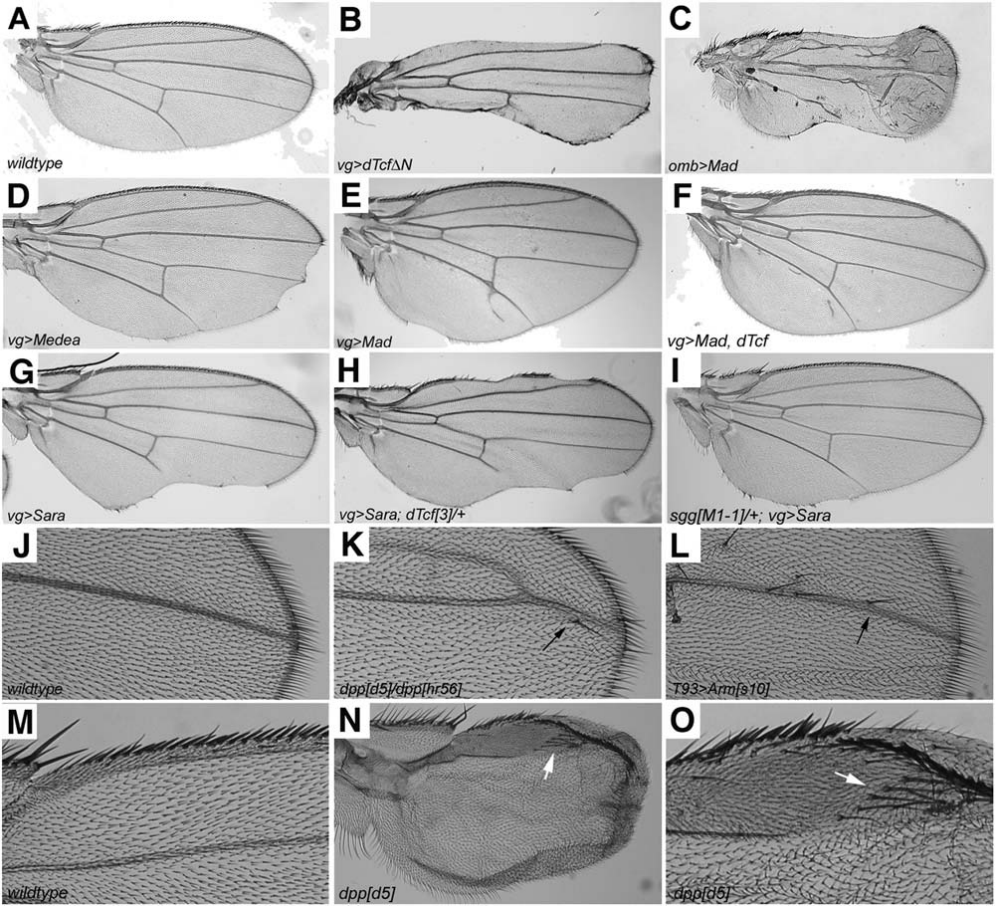
Modulation of BMP signaling in the wing results in *wg*-like phenotypes. (A) Wildtype adult wing. (B) Inhibition of Wg signaling by expression of *vg>dTcfΔN* caused extensive wing notching. Ectopic expression of *omb>Mad* (C), *vg>Medea* (D), *vg>Mad* (E) and the positive regulator of Dpp, Sara, in *vg>Sara* (G) resulted in wing notching. Co-expression of Mad and dTcf with *vg-Gal4* suppressed the wing notches (F). Heterozygosity for *dTcf^3^* enhanced the *vg>Sara* notching (H) while heterozygosity for *sgg^M1-1^* suppressed the notching (I). (J) The wildtype distal portion of the third longitudinal vein (L3). Loss of function transheterozygous alleles of *dpp*, *dpp^d5^/dpp^hr56^* show ectopic bristles (K) that phenocopy ectopic Wg signaling seen in *T93>Arm^s10^* (L). (M) An enlarged view of a wildtype proximal anterior wing margin, showing the normal pattern of bristles. (N) Wing from a *dpp^d5^* adult that lacks most veins, and displays ectopic bristles (arrow). (O) A higher magnification of the patch of extra bristles (arrow) seen in (N).

Previous studies have shown that inhibition of *wg* signaling during wing disc development results in adult wing margin notches of varying severities [14]. For example, expression of a dominant negative form of *dTcf (dTcfΔN)* [3] using the Gal4/UAS ectopic expression system [31] causes a severe loss of anterior and posterior wing margins through inhibition of Wg signaling (Fig. 1B). We have found that ectopic expression of core components of the BMP pathway with various Gal4 drivers also causes similar defects and phenocopied loss of Wg signaling. Expression of *UAS-Mad* with either *omb-Gal4* (Fig. 1C) or *vg-Gal4* (Fig. 1E) or *UAS-Med* with *vg-Gal4* (Fig. 1D), or the activating regulator of BMP, *UAS-Sara,* with *vg-Gal4* (Fig. 1G) in wing discs all caused variable notching of the wing. Bennett et al. (2002) also describe increased wing notching as a consequence of progressively higher levels of BMP signaling [27]. Others have also found that Med and Mad induce such phenotypes upon misexpression, and these effects may involve enhanced stabilization of endogenous Mad [32].

**Figure 2:**
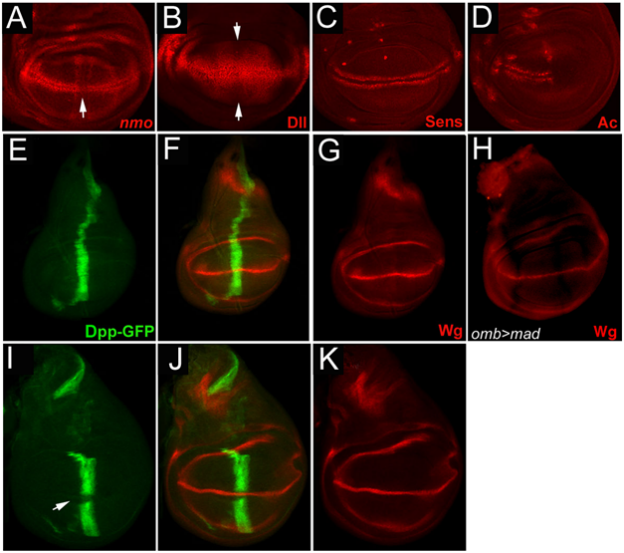
Domains of Wg-target gene expression in vivo. (A–D) Wildtype expression patterns of the four examined Wg targets: (A) *nmo*, (B) Dll, (C) Sens and (D) Ac. The expression of *nmo-lacZ* and Dll are weaker along the A/P boundary, as indicated by arrows in A and B. (E–G) Expression domain of Dpp in the early 3^rd^ instar larvae: In early 3^rd^ instar discs Dpp expression is continuous along the A/P boundary (E) and intersects with the domain of Wg expression (F–G). This localization provides an opportunity for Dpp to affect the expression of the early Wg targets such as *nmo* and Dll in areas of highest Dpp activity (arrows in A and B). The endogenous expression patterns of *nmo* and Dll along the A/P boundary are slightly suppressed relative to the rest of the expression domain, suggesting that *in vivo* endogenous Dpp plays a role in fine-tuning the expression of these Wg target genes (arrows in A and B). Expression domains of Dpp (as seen by expression of *UAS-Dpp-GFP* with *dpp-Gal4*) and Wg intersect in early third instar wing discs (E–G) while later wing discs show a discontinuity of Dpp (I–K, arrow in I). In late 3^rd^ instar discs, the expression of Dpp is suppressed at the D/V boundary (I–K) due to the action of the Notch pathway [20], and thus fails to intersect the highest domain of Wg expression. The absence of an intersection between Wg and Dpp domains may explain the continuous expression of Sens in this region (C). In contrast to the leg disc, ectopic Dpp signalling within the wing pouch using *omb-Gal4>UAS-mad* does not repress endogenous Wg ligand (H).

To further investigate whether Dpp can influence the signaling capacity of the Wg pathway, genetic interaction studies were undertaken. We observe that co-expression of *vg>Mad* and *Tcf* can suppress posterior notches caused by expression of *vg>Mad* alone (Fig. 1F). Consistently, we found that the *vg>Sara-*induced notching (Fig. 1G) was enhanced by heterozygosity for *dTcf^3^* (Fig. 1H) and suppressed by heterozygosity for the Wg inhibitor *sgg^M1-1^* (*zw3/GSK3β*) (Fig. 1I). These interactions suggest the *vg>Sara*-induced notching was due to reduced Wg signaling, and that elevated BMP can inhibit endogenous Wg signaling. This effect is distinct from what is observed in the leg disc (see Introduction) and is not due to the suppression of *wg*, as ectopic BMP signaling does not affect *wg* ligand expression in the wing pouch (Fig. 2H) [24], [25].

### Dpp loss of function has phenotypes associated with Wg gain of function

To further characterize the inhibition of Wg by BMP pathway components, we determined whether *dpp* loss of function mutants display any phenotypes suggestive of elevated Wg signaling. We found that *dpp^d5^/dpp^hr56^* flies displayed ectopic bristles along the L3 vein with 47% penetrance (n = 122; arrow in Fig. 1K). Ectopic bristles were also seen upon expression of activated *UAS-Arm^S10^* with *T93-Gal4* (Figs. 1L) and these are known to be caused by elevated Wg signaling [14], [33]. In addition, rare homozygous *dpp^d5^* flies had tiny wings lacking most vein tissue that displayed patches of ectopic bristles suggesting elevated Wg activity (arrows in Fig. 1N, O).

### Wg target gene expression is inhibited by Dpp signaling

**Figure 3:**
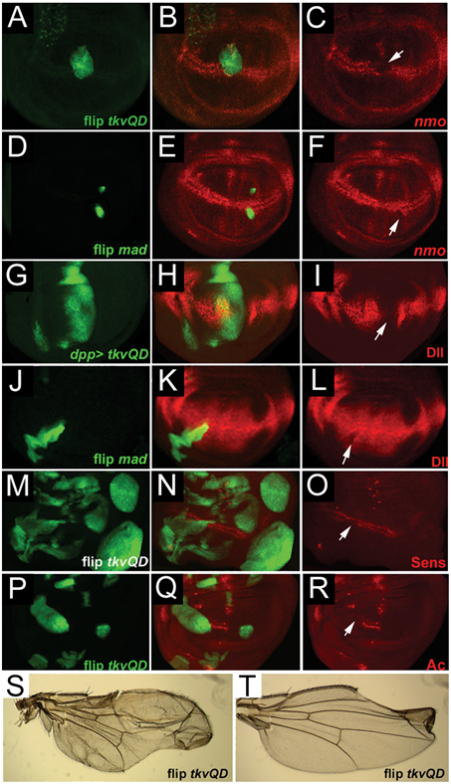
BMP signaling can inhibit Wg-target gene expression *in vivo.* (A–R) Dpp signaling was activated ectopically in 3^rd^ instar wing discs and the expression of Wg target genes was examined. *nmo-lacZ* (as detected by anti-βgal antibody, red in B, C) is suppressed in *UAS-Tkv^QD^* flip out clones (A–C; clone is marked by GFP in A, B; arrow in C) and *UAS-mad* flip out clones (D–F; arrow in F). The arrowhead in C points to the *nmo* expression in vein primordia (15). (G–I) Dll protein expression is suppressed (arrow in I) by *dpp-Gal4>UAS-Tkv^QD^, UAS-GFP* and in *UAS-mad* flip out clones (J–L). (M–O) Sens expression is suppressed (arrow in O) in *UAS-Tkv^QD^* clones. (P–R) Ac expression is suppressed in *UAS-Tkv^QD^* flip out clones (arrow in R). (S, T) Adult wing phenotypes derived from larvae in which flip-out *UAS-Tkv^QD^* clones were induced show inhibition of Wg signaling and Wg target gene expression.

We next examined the expression of four Wg targets, *nemo (nmo)* (Fig. 2A), *dll* (Fig. 2B *), sens* (Fig. 2C) *and ac* (Fig. 2D), in wing discs where the Dpp pathway was activated [10], [14], [34], [35], [36]. We wanted to determine whether the observed adult wing phenotypes and genetic interactions reflected changes in Wg target genes. The flip-out clone technique was used to express either *UAS-Mad* (Fig. 3D, J) or an activated form of the receptor *UAS-Tkv^QD^* (Fig. 3A, M, P) in GFP-marked clones [34], [37]. We obtained similar results from both transgenes, indicating that in this context, expression of high levels of Mad can lead to high levels of BMP pathway activity. In all cases, flip-out clones showed reduced Wg target gene expression (Fig. 3C, F, L, O, R). Expressing *UAS-Tkv^QD^* in the *dpp* expression domain also suppressed Dll protein expression (Fig. 3G–I). Consistent with the disc data, we observed that surviving adults from flip-out *UAS-Tkv^QD^* crosses displayed margin notching, confirming that reduction of target gene expression in larval imaginal discs results in *wg* loss of function adult phenotypes (Fig 3S, T).

### Reduced BMP signaling leads to elevated Wg signaling

**Figure 4:**
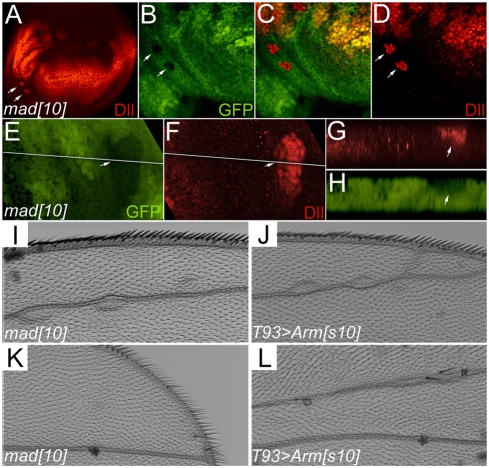
Loss of BMP signaling can induce elevated Wg signaling. *mad^10^* loss of function somatic clones were induced and examined in 3^rd^ instar larval discs and adult. (A) Ectopic Dll is seen in two small clones (arrows). (B–D) Higher magnification of disc in panel A, showing cell autonomous ectopic Dll (red in D) in *mad* clones (marked by arrows and the absence of GFP, green in B). (E, F) A different *mad^10^* clone marked by the absence of GFP (E), showing ectopic Dll (F). (G, H) Z-sections of the regions shown with a white line in panels E and F, revealing the specificity of ectopic Dll to the *mad^10^* clone (arrows). (I–L) Adult wing phenotypes observed after *mad^10^* clone induction (I, K) phenocopy defects observed with ectopic activated Arm (*T93-Gal4>Arms10*) (J, L).

We then sought to demonstrate that an elevation of Wg signaling output is observed upon reduction of BMP signaling. *mad^10^* clones were induced in a *Minute^+^* background and examined for Dll expression. In clones located outside the endogenous Dll domain, in regions of the wing disc exposed to low levels of Wg, a cell autonomous induction of Dll was observed upon loss of *mad* (Fig. 4B–H). Clones within the endogenous Dll domain did not show elevated Dll staining, likely due to saturation of Wg signaling within the Dll domain. Furthermore, as described above, the adult wing phenotypes observed after *mad^10^* clone induction (Fig. 4I, K) closely resemble phenotypes observed with ectopic stabilized Arm (Fig. 4J, L). These observations reveal that in the absence of Mad, Wg target gene expression can be elevated. Thus both increased and decreased Mad signaling can modulate the extent of Wg pathway activity.

### In vitro competition affects Wg-dependent gene expression

Our genetic interaction studies suggest an inhibitory interaction in the wing between the signaling effectors of the Wg and BMP pathways. Specifically, elevating the levels of BMP signal through the ectopic expression of Mad or activated Tkv led to diminished expression of Wg targets. Since it has been shown previously in vertebrate as well as Drosophila that members of the Lef/Tcf family of proteins can associate with Smads, we sought to investigate the possibility that sequestering of dTcf by Mad in the wing could lead to a reduction in Wg signaling output.

**Figure 5:**
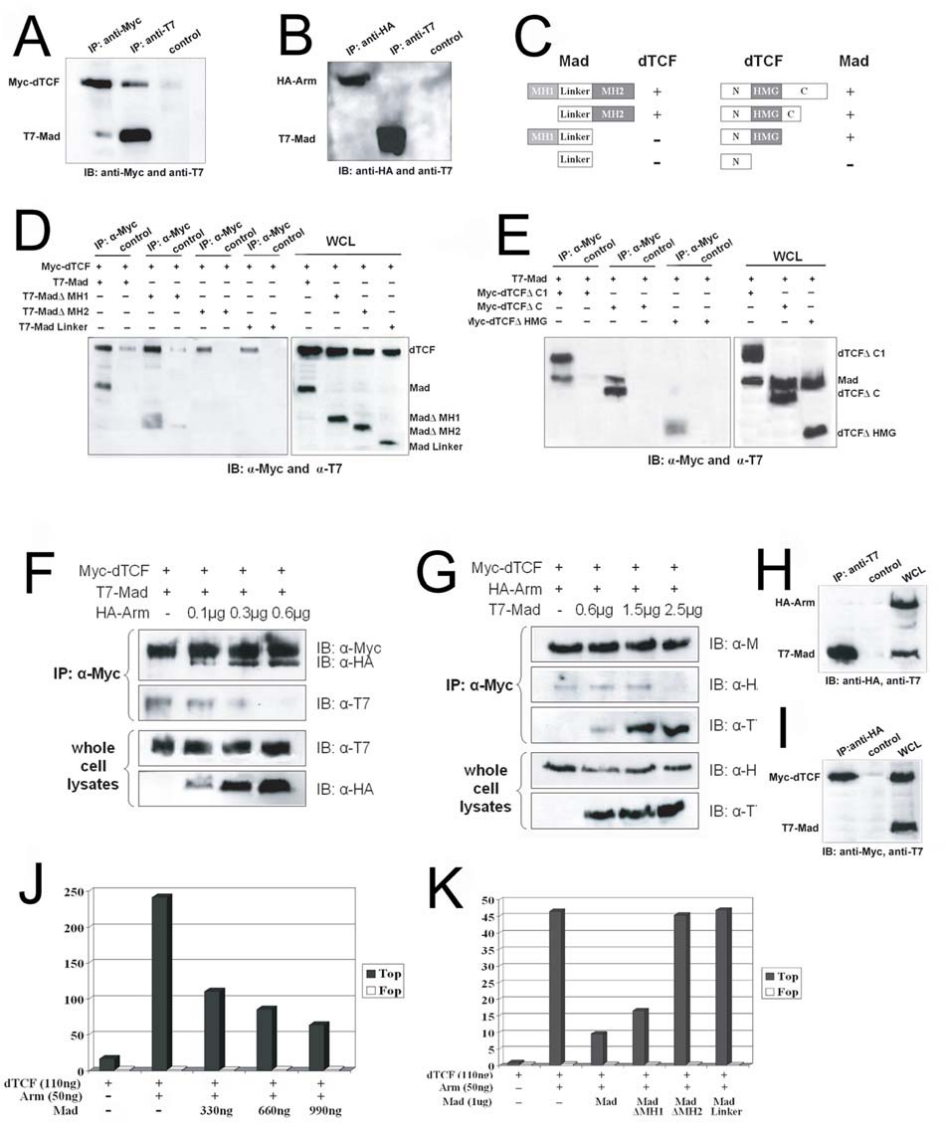
Mad and dTcf form a complex that competes with Arm-dTcf binding and blocks dTcf-dependent gene expression. (A) Binding of Mad and dTcf. pCMV-T7-Mad and pCMV-Myc-dTcf were co-transfected into HEK293 cells. Cell lysates were immunoprecipitated (IP'd) with anti-Myc, anti-T7 or IgG (control). Immunoblotting (IB) was performed with anti-Myc and anti-T7 antibodies. (B) Mad and Arm do not associate directly. pCMV-T7-Mad and pCMV-HA-Arm were co-transfected into HEK293 cells. Cell lysates were IP'd with anti-HA, anti-T7 or IgG (control). IB was performed with anti-HA and anti-T7 antibodies. (C) A schematic map of the dTcf and Mad truncation constructs and indication of their ability to bind the other. (D) dTcf binds the MH2 domain of Mad. HEK293 cells were transfected with Myc-dTcf and the indicated T7-Mad constructs. Cell lysates were IP'd with anti-Myc or IgG (control). IB was performed with anti-Myc and anti-T7 antibodies. (E) Mad interacts with the HMG domain of dTcf. HEK293 cells were transfected with T7-Mad and Myc-dTcf constructs. Cell lysates were IP'd with anti-Myc or IgG (control). IB was performed with anti-Myc and anti-T7 antibodies. WCL, whole cell lysates. (F) Increasing concentrations of Arm can inhibit the Mad/dTcf complex. 1.5 mg of T7-Mad, 1.5 mg of Myc-dTcf and increasing amounts of HA-Arm were co-transfected into HEK293 cells. Cell lysates were IP'd with anti-Myc. IB was performed with anti-HA, anti-Myc and anti-T7 antibodies. (G) High concentrations of Mad can inhibit Arm/dTcf complex formation. 500 ng of HA-Arm, 800 ng of Myc-dTcf and increasing amounts of T7-Mad were co-transfected into HEK293 cells. Cell lysates were IP'd with anti-Myc. IB was performed with anti-HA, anti-Myc and anti-T7 antibodies. (H) Transfected cell lysates expressing HA-Arm, Myc-dTCF and T7-Mad were IP'd with anti-T7, followed by IB with anti-T7 and anti-HA, showing that Mad did not bind to Arm (I) Transfected cell lysates expressing HA-Arm, Myc-dTCF and T7-Mad were IP'd with anti-HA, followed by IB with anti-T7 and anti-Myc, showing that Arm did not pull down Mad. (J) Topflash assays in HEK293 cells showed inhibition of dTcf/Arm-dependent transcription by Mad. Topflash values are indicated on the left. These values are from the average of three independent transfection experiments. Vectors used for each experiment are as indicated in the figure. The negative control Fopflash values are given on the right in white columns. (K) Only Mad forms that can bind dTcf can inhibit Topflash expression, indicating the interaction must be direct.

To further characterize the mechanism of Wg inhibition by BMP signaling, biochemical studies were performed with dTcf, Arm and Mad. Immunoprecipitations (IPs) were performed from HEK293 cells transfected with Drosophila expression constructs. These experiments showed an interaction between Mad and dTcf (Fig. 5A), but not between Mad and Arm (Fig. 5B). Next, Mad and dTcf binding domains were mapped using truncation constructs (Fig. 5C). Mad truncations were made in which the two conserved MH1 and MH2 domains were deleted. The MH1 domain contains the DNA binding domain, while the MH2 domain is involved in protein-protein interactions and transcriptional activation (reviewed in [38]). dTcf can bind full length Mad and MadΔMH1, but not MadΔMH2 or Mad-linker, thus dTcf binds the MH2 domain of Mad (Fig. 5D). Mad binds two C-terminal truncations of dTcf, but not a deletion of the HMG domain (Fig. 5E), indicating that Mad binds the DNA-binding HMG domain of dTcf.

To address whether the binding of Mad and dTcf affects the Arm/dTcf complex, protein binding was examined in cells triply transfected with Mad and dTcf and increasing amounts of Arm. dTcf precipitated both Mad and Arm when the Arm amount was relatively low (Fig. 5F, lanes 2 and 3), while increasing amounts of Arm blocked the binding of dTcf and Mad in a dose-sensitive manner (Fig. 5F, lane 4). Reciprocally, cells were transfected with dTcf, Arm and increasing amounts of Mad (Fig. 5G). Mad, dTcf and Arm were co-immunoprecipitated under conditions in which the Mad amount was relatively low (Fig. 5G, lanes 2 and 3), but higher levels of Mad blocked the Arm/dTcf complex (Fig. 5G, lane 4). Since dTcf can bind both Mad and Arm, we examined whether the proteins form a heterotrimeric complex. When lysates from cells expressing all three proteins were immunoprecipitated (IP), a Mad IP (anti T7) failed to pull down Arm (Fig. 5H) and an Arm IP (anti HA) failed to pull down Mad (Fig. 5I), suggesting that the precipitates seen in Fig. 5F, G (lanes 2, 3) represent mutually exclusive complexes of dTcf/Arm and dTcf/Mad.

### High levels of Mad can inhibit Wg-dependent gene expression in vitro

To study the effect on transcription of Mad/dTcf binding, the Tcf-responsive Topflash reporter was used (Fig. 5J, K) [39]. Co-transfection of Arm and dTcf abundantly induced Topflash (Fig. 5J, K). Co-transfection with full length Mad caused a dose-sensitive inhibition (Fig. 5J). Transfection of MadΔMH2 or the Mad linker did not inhibit Topflash expression, showing that binding between Mad and dTcf was required for the inhibition (Fig. 5K). MadΔMH1 could inhibit Topflash, but not to the degree that full length Mad could, indicating that some inhibitory function is retained in the MH1 domain. Thus, expression of forms of Mad that can bind dTcf resulted in a decrease in Wg-dependent gene expression.

### In vivo competition

**Figure 6:**
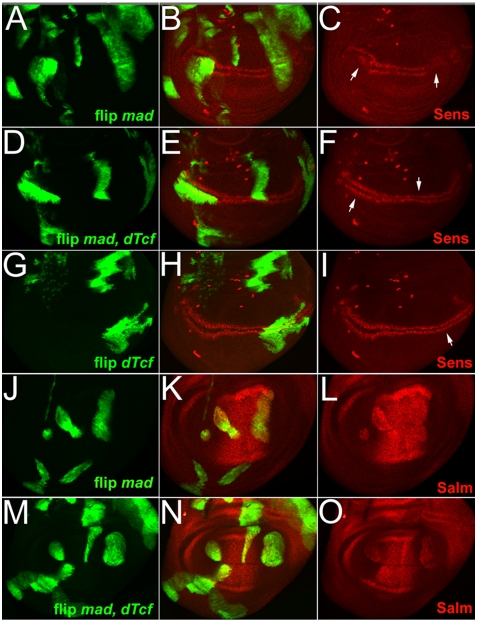
In vivo competition between dTcf and Mad affects Wg target genes. Flip-out clones (positively marked with GFP) were generated to express Mad and/or dTcf. (A–C) Sens expression was suppressed in Mad misexpression clones (arrows in C). (D–F) No reduction of Sens was seen in double flip-out clones expressing Mad and dTcf (arrows in F). (G–I) Flip-out dTcf clones showed no reductions in Sens (arrows in I). (J–L) Flip-out Mad clones induced ectopic expression of the Dpp target Salm. (M–O) Double flip-out clones expressing Mad and dTcf did not show suppression of the ectopic induction of Salm.

To test the hypothesis that excess Mad can saturate dTcf in vivo, Wg target gene expression was monitored in wing discs clones ectopically expressing Mad and dTcf. Our prediction would be that Mad inhibits Wg targets by competing with Arm for dTcf binding. Thus, if excess dTcf is provided, it should alleviate the repressive effect of Mad and allow dTcf/Arm-driven transcription to proceed. Ectopic dTcf in flip-out clones showed no change in Sens expression (Fig. 6G–I), consistent with the lack of phenotype seen with *vg>dTcf* expression. Ectopic expression of dTcf does not lead to a modulation of transcription as members of the Lef/Tcf family of transcription factors are abundantly expressed and bound to DNA and must rely on association with co-factors to activate gene transcription (reviewed in [1]). On the other hand, as shown previously in Fig. 3, flip-out Mad clones showed suppressed Sens expression (Fig. 6A–C). Simultaneous expression of dTcf in such clones blocked the inhibition caused by Mad and the normal expression pattern was seen (Fig. 6D–F). Similar results were obtained for the expression of Dll and *nmo* (data not shown). Thus, enhanced levels of dTcf could suppress the negative effects of ectopic Mad on Wg transcriptional output. These observations strengthen our model in which ectopic Mad competes with dTcf and leads to a reduction in Wg signaling output. By expressing even higher levels of dTcf, we effectively were able to titrate the suppressive effects of elevated Mad protein.

To determine if the effect we observed was specific to Wg target genes, we examined the expression of the Mad target gene *spalt major* (*salm*) [40]. Flip-out Mad clones showed ectopic Salm protein (Fig. 6J–L). This gene activation was not suppressed by the simultaneous expression of dTcf (Fig. 6M–O) suggesting that the interaction of Mad and dTcf specifically blocks dTcf-dependent transcription.

## Discussion

In this study, we show that Wg-dependent gene expression can be modulated in vivo by elevated BMP signaling due to activated receptor or high levels of Mad. We find that the molecular basis for this effect arises through Mad/dTcf complex formation, which can inhibit the binding of Arm with dTcf and block Wg-dependent gene expression in vitro. We propose that Mad and Arm compete for binding of dTcf, and that ectopic nuclear Mad inhibits Wg signaling through direct binding with dTcf. In support of this model, overexpression of dTcf inhibits Mad-dependent suppression of Wg target gene expression in vivo. Thus elevated Dpp signaling can inhibit Wg signaling both in vitro and in vivo. We also show that loss of BMP signaling can result in elevated Wg target gene expression, suggesting the interaction between the two pathways normally acts to fine-tune the Wg response.

Consistent with our findings, Takaesu et al. (2005) describe that expression of a dominant negative human Smad4 construct in Drosophila wings leads to elevated Wg signaling and target gene expression [41]. The molecular mechanism of this interaction is not yet known, but may involve mutant Smad4 titrating endogenous Mad protein, thus mimicking our *mad* loss of function studies.

We and others [27], [32] have shown that ectopic expression of Mad or Med generates wing margin notches, which mimic a loss of Wg phenotype. Adachi-Yamada et al. (1999) reported that overexpression of a constitutively active version of Tkv leads to activation of the JNK apoptotic pathway and the consequence is loss of wing tissue [42]. We cannot exclude the possibility that either *vg>Mad* or *vg>Med* may activate the cell death pathway directly. However, the observation that the notching phenotype can be enhanced by loss of *dTcf* and rescued by gain of Wg, as seen with the *zw3^+/−^* heterozygous mutant, supports the hypothesis that *vg>Mad* and *vg>Med* suppress Wg signaling activities, therefore leading to the terminal consequence: apoptosis in the wing margin. Indeed, reduction of Wg signaling leading to activation of the JNK apoptotic pathway has been elegantly illustrated by Giraldez and Cohen (2003)[43].

We have shown that both *dpp* loss of function mutants and *mad* somatic clones display ectopic bristles phenotypes on the wing blade, a phenotype indicative of elevated Wg signaling. The relatively weak adult phenotype can be explained if Dpp attenuation of Wg signaling plays a fine-tuning role in a specific developmental time window. Likely the damage caused by loss of *dpp* and *mad* can be compensated for by later development events. During early larval development, Dpp and Wg execute their global patterning function in organizing the A/P and D/V axes. In this stage, Dpp signaling antagonizes Wg signaling at the A/P-D/V intersection. In the late 3^rd^ instar larval and pupal stages, Wg functions in wing margin organization, which requires repression of *dpp* expression [44]. Consequently, in this stage Dpp signaling is not active in the wing margin. Therefore, suppression of the Dpp pathway does not lead to a severe loss of the wing margin, and only rare cases of ectopic bristle phenotype were observed in the Dpp signaling mutants. However, overexpression of Dpp signaling in this stage can cause loss of the margin (Figure 1C–F, Fig 3 S, T). In support of the notion, we found that in the early larval stage, Dpp and Wg domains intersect, suggesting a possible crosstalk of the two pathways (Fig 2E–G). In the late 3^rd^ instar, Dpp expression is suppressed when the A/P and D/V boundaries intersect (Fig 2I–K; [44]). Taken together, we postulate that the mechanism of Dpp inhibiting Wg signaling is temporal and likely functions in the early larval stage to fine-tune the global patterning of the wing disc.

Nevertheless, we observe that altering the levels of BMP signaling is sufficient to modify Wg target gene expression. We observed ectopic Dll in *mad* mutant clones, and suppression of Dll, *nmo*, Sens, and Ac expression upon overexpression of *mad* and in activated *tkv* flp-out clones.

Several studies in vertebrate have shown that association between Smad and Lef/Tcf plays significant roles in controlling certain developmental events. In Xenopus, Smad interacts with Lef1 to synergistically activate *Xtwn* transcription [16], [17]. In mice, a Smad- Lef/Tcf complex is implicated in transcription of *c-myc*, *Emx2*, *MSX2*, and *gastrin* [18], [45], [46], [47]. These studies suggest a general molecular mechanism that not only is the Smad-Lef/Tcf complex required, but also the promoter specific cis-elements are needed for the synergistic activation of the target genes. For example, activation of *Xtwn* requires Smad3 binding to the Smad binding elements (SBEs); *c-myc* activation needs Smad4 binding to the Lef/Tcf binding elements 1 (TBE1). Our studies in the fly wing unveil a novel mechanism of interaction of the Smad-Tcf complex, in which it exerts an antagonistic role on Wg target gene expression, both *in vivo* and *in vitro.* The antagonism is *cis*-element independent, as evidenced by the finding that expression of Mad *in vitro* can inhibit Tcf-response in Topflash assays, where reporter gene expression is controlled solely by Tcf binding sites. These in vitro findings are consistent with the modified levels of targets we observe in the wing, namely Dll, *nmo,* Sens and Ac. Our biochemical studies suggest a more general molecular mechanism for the Smad-Lef/Tcf interaction in the wing, in which Mad and Arm compete for the binding of dTcf. Excessive Mad can inhibit the association of Arm/dTcf *in vitro.* The suppression of Wg target genes by ectopic Mad can be rescued by replenishing the dTcf pool (co-expression of dTcf) *in vivo*. Although a different molecular mechanism is proposed here, the binding domains between Smad and Tcf are conserved between Drosophila and vertebrate. Similar to the vertebrate study [16], our study indicates that the MH2 domain of Mad associates with the HMG domain of dTcf. Previous research showed that the amino terminus of dTcf binds to Arm [3]. The fact that Mad and Arm interact with different domain of dTcf independently would not exclude the possibility of competition for binding, due to conformational changes upon protein/protein interaction. It is intriguing to speculate that such a Smad- Lef/Tcf antagonism is also conserved in vertebrates.

Distinct tissue-specific mechanisms of interaction between Wg and BMP signaling have evolved. For example, in contrast to the mutual repression of Wg and BMP seen in leg discs, elevated Dpp actually induces *wg* expression during gut development [48]. Our study describes an antagonism that acts to fine-tune the level of Wg signaling in the wing pouch through competition between Mad and Arm for dTcf binding. We propose that the different expression domains, tissue specific regulators and temporal patterns of activation will determine the specificity of the different modes of regulation. The interaction we observe in the wing represents a novel mechanism of interaction between Wg and BMP signaling and highlights the importance of cross regulation of signaling pathways during development.

## Materials and Methods

### Fly strains

The following fly strains were used: *nmo-lacZ (nmo^P^*) [43], *UAS-lacZ, UAS-Mad*, *UAS-Med*, *UAS-Tkv^QD^* [37], *UAS-Sara^F678A^* (this form of Sara leads to elevated signaling by blocking receptor downregulation) [27], *vg-Gal4* (expressed in the wing pouch), *omb-Gal4* (expressed along the D/V boundary), *T93-Gal4*, *UAS-Arm^s10^* [49], *UAS-dTcf*, *dTcf^3^* and *UAS-dTcfΔN* [3], *Ay-Gal4.25-UAS-GFP.S65T*, *dpp^d5^*, *dpp^hr56^*, *sgg^M1-1^*and *mad^10^* [50].

### Flip-out clones, somatic loss of function clones and antibody staining

Flip-out ectopic expression clones and staining were generated as described in [34], [51]. For each genotype, at least 30 clones were examined. Somatic *mad* clones were generated by crossing *hsflp.22/Y; M(2)21AB^1^ GFP FRT40A/CyO* males to *yw; mad^10^ FRT40A/In(2LR) Gla* females. Embryos were collected for 24 hours and heat-shocked at 38°C for 90 minutes at 72–96 hours after egg laying. *mad^10^* clones located outside of the endogenous Dll domain were examined and ectopic Dll was observed in 39% (n = 33).

The following antibody dilutions were used: rabbit anti-βgal (1∶2000), rat anti-Dll (1∶500), mouse anti-Dll (1∶400)[52], mouse anti-Ac (1∶50), rabbit anti-GFP (1∶1000), rabbit anti-Salm (1∶600) and guinea pig anti-Sens (1∶1500), anti-Wg (1∶100).

### Expression vectors

pCMV-HA-Arm and pCMV-Myc-dTcf were generated by D. Bessette (D.B. and E.M.V., unpublished). dTcf constructs were generated in pCMV-Myc. The dTcfΔC1 construct encodes amino acids (a.a.) 1–522 (full length dTcf is 751 a.a.); dTcfΔC encodes a.a. 1–394; dTcfΔHMG encodes a.a. 1–244. Mad constructs were generated from pCMV-T7-Mad [48]. MadΔMH1 encodes a.a. 157–455; MadΔMH2 encodes a.a. 1–256; Mad linker encodes a.a. 157–256. More details on construct generation can be supplied upon request. Co-IP's were performed using standard protocals.

### Topflash reporter assay

HEK293 cells were cultured in 6 well plates and transiently transfected by using Polyfect (Qiagen). The renilla luciferase pRL-CMV served as an internal control. Transfections contained 1 mg of pTOPFLASH reporter, 0.1 mg of pRL-CMV and others as described in Figs. 3J, K. pCMV empty vector was used to add to a total of 1.15 mg per well. Luciferase assays were performed with the Dual Luciferase Reporter assay system (Promega) according to the manufacturer's instructions and as described in Korinek et al. (1997). Each experimental condition was examined three times and the results were standardized against the internal controls.
